# Don’t Count Your Chicken Livers: an Outbreak of <i>Campylobacter</i> sp. Not Associated with Chicken Liver Parfait, England, November 2013

**DOI:** 10.1371/currents.outbreaks.c1b19bae7bac20dccf00ef18b19d8d2a

**Published:** 2014-08-12

**Authors:** Suzan Trienekens, Charlotte Anderson, Jennifer Duffy, Rachel Gill, Lisa Harvey-Vince, Helen Jones, Piers Mook, Chikwe Ihekweazu, Ishani Kar-Purkayastha

**Affiliations:** Field Epidemiology Services – Victoria Office, Public Health England, London, UK; Field Epidemiology Services – Victoria Office, Public Health England, London, UK; Surrey & Sussex Health Protection Team, Public Health England, Horsham, UK; Surrey & Sussex Health Protection Team, Public Health England, Horsham, UK; Surrey & Sussex Health Protection Team, Public Health England, Horsham, UK; Environmental Health – Runnymede Borough Council, Addlestone, UK; Field Epidemiology Services – Victoria Office, Public Health England, London, UK; Field Epidemiology Services – Victoria Office, Public Health England, London, UK; Surrey & Sussex Health Protection Team, Public Health England, Horsham, UK

**Keywords:** campylobacter, disease outbreaks, Epidemiology, food-borne infection

## Abstract

In England, several recent campylobacter outbreaks have been associated with poultry liver consumption. Following a lunch event in a hotel in Surrey in November 2013 where chicken liver parfait was served, guests reported having gastrointestinal symptoms. A retrospective cohort study showed 46 of 138 guests became unwell, with a median incubation period of two days and for 11 cases campylobacter infection was laboratory confirmed. Food item analysis identified an association between illness and consumption of roast turkey (aOR=3.02 p=0.041) or jus (aOR=3.55 p=0.045), but not with chicken liver parfait (OR=0.39 p=0.405). The environmental risk assessment did not identify non-compliance with standard food practice guidelines. This study presents a point-source outbreak of campylobacter with a high attack rate and epidemiological analysis results show that the jus or roast turkey was the likely source of infection although this could not be confirmed by the environmental assessment. Consuming the chicken liver dish was not a risk factor for developing symptoms as was initially hypothesised. Prior knowledge on the association between poultry liver food items and campylobacter outbreaks should not overly influence an outbreak investigation to ensure the true aetiology is identified and on-going public health risk is minimised.

## Introduction **

Campylobacter remains the most commonly reported cause of foodborne infections in England and Wales, and reports have increased from 48,133 in 2002 to 65,032 laboratory confirmed cases in 2012.^1^ Outbreaks of campylobacter have often been associated with poultry liver consumption in recent years in the UK 2 ^,^ 3 ^,^ 4 ^,^ 5 ^,^ 6 ^,^ 7 and elsewhere, including Australia 8 and the United States 9 , mainly due to undercooking of the meat. 10 A UK study from 2011 showed that prevalence of campylobacter from retail chicken liver was 81% and that a high number of strains found were also commonly found in humans. 11 The proportion of campylobacter outbreaks linked to poultry liver consumption in the UK ranged from 72-85% between 2007 and 2012 and was 44% in 2013, with 180 confirmed cases reported. 12 [Public Health England (PHE), personal communication]

On 2 December 2013, Surrey and Sussex Health Protection Team (SSHPT) was alerted by the Environmental Health Officer at Runnymede Borough Council to 14 reports of gastrointestinal illness among attendees of a company lunch event. This event was held at a hotel in Surrey on 21 November 2013, and was attended by 138 guests. SSHPT convened an outbreak control team (OCT) including the Field Epidemiology Service of PHE and Environmental Health Officers from the council and the OCT agreed to conduct an outbreak investigation.

The aims of this study were to describe the outbreak in place, person and time; the number of ill among attendees; symptoms and severity of illness; and to identify possible risk factors associated with illness. This paper presents the epidemiological, environmental and microbiological findings of the study.

## Methodology

Epidemiological study

A retrospective cohort study was conducted among the 138 guests of the lunch event. A case was defined as “An individual who attended and ate at the lunch event at the hotel on 21 November 2013 and became unwell with diarrhoea and/or vomiting with symptom onset between 21 November and 1 December 2013”. Attendees having gastro-intestinal symptoms during any of the seven days prior to the event were excluded from the study. The event organiser provided the complete guest list and corresponding contact details, which made the cohort design feasible. The menu was provided by the hotel.

Information on demographics, gastrointestinal symptoms and severity of illness and food exposures were collected using a structured online questionnaire. A link to the questionnaire was emailed to attendees for whom an email address was available on 6 December. If an email address was not available, guests were contacted by telephone to obtain an email address or orally administer the questionnaire. All subjects provided informed consent.

Data management, validation and analysis were performed using Microsoft Excel and Stata 12.0. An epidemiological curve showing the distribution of symptom onset was produced. Characteristics of cases and non-cases were described in time, place and person and compared using t-tests for continuous variables and chi square tests or Fisher’s exact tests, as appropriate, for categorical variables. As information was not available for the complete cohort, odds ratios instead of relative risks were calculated to avoid possible bias. Odds ratios for each food and drink item were calculated using logistic regression and exact logistic regression when an odds ratio could not be derived. Variables associated with an increased odds of illness at p≤0.2 in the univariable analysis, along with age (as an a priori confounder), were analysed further using stepwise backwards logistic regression. Variables were retained if they were independent risk factors for illness, or improved the fit (tested using the Likelihood Ratio Test), and adjusted odds ratios calculated. Dose-response relationship with doses categorised as ‘small portion’, ‘most of the portion’ or ‘whole or more than a portion’ was assessed for variables associated with illness.

Microbiological investigation

Environmental investigation at the venue was undertaken following the initial report of the outbreak. Symptomatic attendees who visited their GP submitted stool specimens which were tested for common food-borne pathogens. Unknown results or results not available at the time of completing the questionnaire were followed up by contacting general practices.

Environmental risk assessment

Environmental investigations focused on assessing food handling practices at three stages: following the initial report, after the first OCT meeting and to follow-up on the findings of the epidemiological study. These investigations included an audit of all cooking and re-heating temperatures and processes, inspection of cooking appliances and a general assessment of the kitchen, toilet facilities and general site. The Environmental Health Officer also asked the venue about any staff illness and whether other reports of illness amongst other diners were reported.

## Results

Epidemiology

Of the 138 attendees, valid questionnaire responses from 102 were obtained for analysis (response rate: 74%). From these, 70 questionnaires were completed online and 32 by telephone.

The study population was predominantly male (97%) and the median age was 65 (range: 28-90). Forty-six respondents were classified as cases, an attack rate of 45% of the study population and a minimum of 33% of all attendees of the lunch event. The epidemiological curve was consistent with a point source exposure (Figure 1). Illness peaked between 2-3 days after exposure, with the first person becoming ill a few hours after the lunch and the last person becoming ill on 29 November, eight days after the lunch.

**Epicurve with date and time of onset of cases (n=42):**
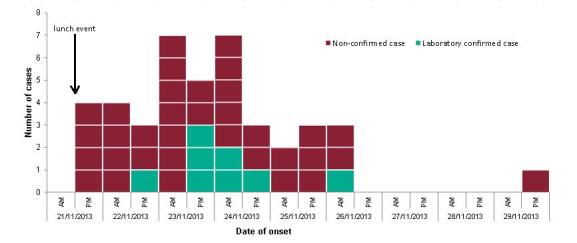
Footnote: Four cases did not report a time of onset: two on 22/11/2013, and two (one confirmed case) on 24/11/2013

Table 1 presents demographics and illness history of the study population. The age of cases ranged from 41-84 years, with a median of 65, and did not differ significantly from the age of the non-cases (p=0.476) (Table 1). All cases were male. Most commonly reported symptoms were diarrhoea (98%), lethargy/tiredness (78%) and abdominal pain (72%). Cases reported feeling unwell for between 3 hours and 23 days at the time of reporting (median: 6 days) (Figure 2). Thirty-nine per cent of the cases sought medical care: eighteen cases visited the GP, and one of these attended a hospital.

**Figure:**
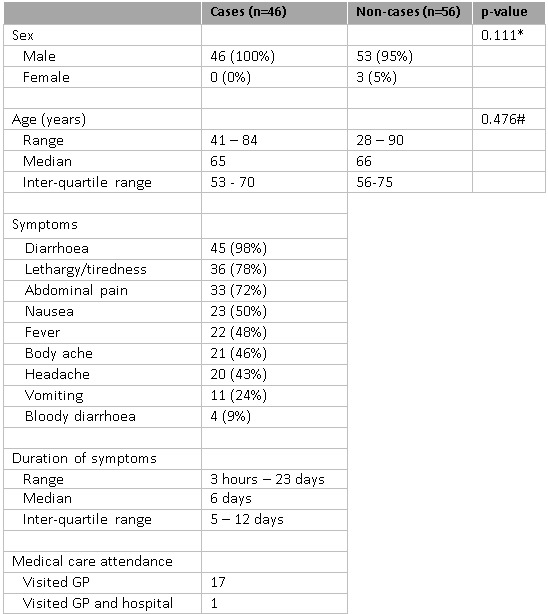
**Table 1: Demographics and illness history (n=102)** *A chi square test was used to compare sex between cases and non-cases #A t-test was used to compare age between cases and non-cases

**Duration of symptoms of cases (n=46):**
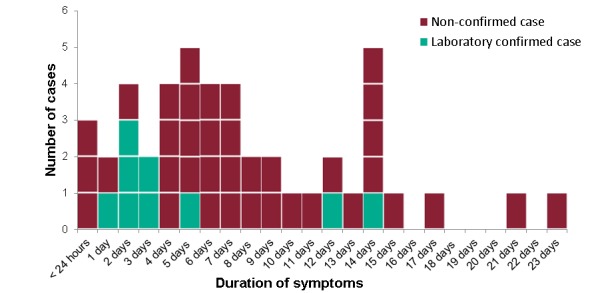
Footnote: Ten cases were still unwell at the time of reporting

Table 2 presents the percentage of cases and non-cases being exposed to each food item and the odds ratio (OR) of becoming ill. The exposures associated with illness were roast turkey (OR 4.54, p=0.125) and the jus (OR 3.28, p=0.028), a highly flavoured thin liquid made with water, wine, bones and vegetables. All cases reported eating the roast turkey and 89% of cases reported eating the jus. Consuming chicken liver parfait was not a risk factor for becoming unwell (OR 0.39, p=0.405). The dose-response analysis did not show any increase in risk of illness with increased portion size for the jus or roast turkey (results not shown).

**Table 2: Univariable and multivariable food and drink analysis (n=102):**
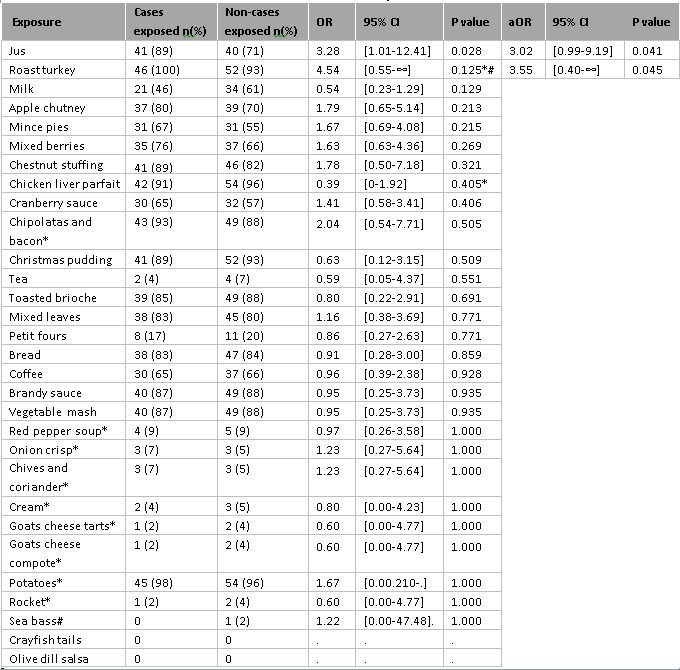
* Fisher’s exact test was used in place of chi square test in these instances. # Odds Ratio calculated using exact logistic regression. OR = Odds Ratio aOR = Adjusted Odds Ratio CI = Confidence Interval

The variables ‘roast turkey’ and ‘jus’ were included in the multivariable model, and remained independent risk factors for illness (Table 2). Controlling for age did not improve the model (continuous variable: p=0.609, categorical variable: 0.779).

Microbiology

Fourteen cases provided a stool sample to their care provider, from which 11 tested positive for campylobacter, although two of these positive cases did not complete a questionnaire. One sample tested negative and two were rejected so no results could be obtained. No further typing was performed.

Environmental assessment

No food samples from the event were available for testing. The premises had attained the highest score (Level 5) on hygiene eight months prior to the outbreak as part of routine inspections by the environmental health department of the local council. All recorded cooking temperatures and practices were compliant with food preparation standards. Kitchen and toilet facilities were found to be well-maintained and no evidence of practice that could lead to on-going public health risk at the restaurant was identified. The turkey and jus were prepared independently in the restaurant. The turkey was raw when delivered to the premises and supplied by a reputable source in the UK. Handling of the raw turkey and further cooking practices were performed by the same chef. The jus was not prepared using bones from the turkey, but with veal bones, which were roasted off with onion, carrot and celery and wine and water were added. Subsequently, the jus was boiled and simmered and thickened with gravy powder. No illness from other parties consuming jus from the same batch was reported passively.

## Discussion

This study describes an outbreak of severe gastrointestinal illness with a high attack rate (46/102) among attendees of a lunch event. The epidemiological curve was consistent with an outbreak caused by a point source exposure. Thirty-nine per cent of the cases sought medical care, and many remained unwell for long periods of time. Symptoms of diarrhoeal illness, an incubation period ranging from a few hours to 8 days and microbiological findings from 11 guests are consistent with infection with campylobacter. Illness was associated with eating roast turkey and jus. An association between illness and eating chicken liver parfait, as was initially hypothesised, was not found. Environmental assessment of the venue found that the various cooking temperatures and practices were in line with standard guidelines and no on-going public health risk was perceived. We were unable to confirm the point of contamination of the two food items identified by the epidemiological investigation. With early confirmation of campylobacter in stool samples, the initial stages of the environmental investigation included detailed analysis of the preparation of the chicken liver parfait. Later investigation focused on detailed analysis of the preparation of the turkey and jus as the questionnaire analysis identified these foods as the most likely source of infection.

Although we achieved a high response rate for a study of this type (74%), we received no information on a quarter of the attendees and there might be further unreported cases. Most questionnaires were filled in online by respondents, but a third of the questionnaires were administered over the phone. To minimise any response bias, we used the same structured questionnaire for both methods. Although public health action was taken rapidly after the initial illness reports, and administering the questionnaire via email instead of using paper questionnaires limited the response period, there were difficulties in food exposure recall for the older study population at the time of completing the questionnaire.

Although previously published studies have found other causes of food-borne campylobacter outbreaks, including consumption of unpasteurised milk 13 ^,^ 14 or raw peas contaminated with wild bird faeces 15, a high proportion of outbreaks have been linked to poultry liver dishes.^12^ In particular chicken liver parfait, which has become a popular dish in England in recent years.

This study shows, however, that an OCT should be wary not to become prejudiced by prior experience, and undertake the investigation of any outbreak with appropriate scientific rigour to ensure where possible that the true cause of illness can be identified and appropriate control measures taken to reduce the risk of further exposure to the public.
